# Supplementary material for: Modeling glioblastoma heterogeneity as a dynamic network of cell states
Source: Mol Syst Biol. 2021 Sep 16;17(9):e10105. doi: 10.15252/msb.202010105 (PMC8444284; doi:10.15252/msb.202010105)
Supplement: Supplementary file 6 — Source Data for Figure 5 [file MSB-17-e10105-s004.zip › Figure5A_sourcedata/GSEA_3071/nosigdb_stateI.GseaPreranked.1622795145664/NB_KI_CLUSTER5.html]

Details for gene set NB\_KI\_CLUSTER5[GSEA]

|  || Dataset | state13071 |
| Phenotype | NoPhenotypeAvailable |
| Upregulated in class | na\_neg |
| GeneSet | NB\_KI\_CLUSTER5 |
| Enrichment Score (ES) | -0.62816674 |
| Normalized Enrichment Score (NES) | -3.1097436 |
| Nominal p-value | 0.0 |
| FDR q-value | 0.0 |
| FWER p-Value | 0.0 |
Table: GSEA Results Summary

  

Fig 1: Enrichment plot: NB\_KI\_CLUSTER5      
 Profile of the Running ES Score & Positions of GeneSet Members on the Rank Ordered List

  

| PROBE | GENE SYMBOL | GENE\_TITLE | RANK IN GENE LIST | RANK METRIC SCORE | RUNNING ES | CORE ENRICHMENT || 1 | CEP152 |  |  | 1446 | -0.253 | -0.4167 | No |
| 2 | GAPDH |  |  | 1459 | -0.253 | -0.4156 | No |
| 3 | BUB3 |  |  | 1687 | -0.278 | -0.4767 | No |
| 4 | MIS18BP1 |  |  | 1764 | -0.289 | -0.4936 | No |
| 5 | RAN |  |  | 1907 | -0.306 | -0.5295 | No |
| 6 | KPNA2 |  |  | 1944 | -0.312 | -0.5343 | No |
| 7 | SPDL1 |  |  | 1968 | -0.314 | -0.5353 | No |
| 8 | CHEK1 |  |  | 2108 | -0.336 | -0.5697 | No |
| 9 | MXD3 |  |  | 2201 | -0.352 | -0.5901 | No |
| 10 | PLK1 |  |  | 2206 | -0.353 | -0.5849 | No |
| 11 | NUCKS1 |  |  | 2278 | -0.368 | -0.5989 | No |
| 12 | TUBB4B |  |  | 2291 | -0.370 | -0.5957 | No |
| 13 | CKS1B |  |  | 2332 | -0.380 | -0.6004 | No |
| 14 | CKAP2 |  |  | 2357 | -0.385 | -0.6004 | No |
| 15 | ZWILCH |  |  | 2358 | -0.385 | -0.5934 | No |
| 16 | CENPW |  |  | 2402 | -0.393 | -0.5988 | No |
| 17 | H2AFV |  |  | 2431 | -0.398 | -0.5997 | No |
| 18 | CKS2 |  |  | 2466 | -0.404 | -0.6023 | No |
| 19 | RACGAP1 |  |  | 2522 | -0.419 | -0.6107 | No |
| 20 | TEX30 |  |  | 2556 | -0.428 | -0.6126 | No |
| 21 | NCAPD2 |  |  | 2563 | -0.431 | -0.6065 | No |
| 22 | H2AFZ |  |  | 2572 | -0.433 | -0.6010 | No |
| 23 | ANP32B |  |  | 2593 | -0.438 | -0.5989 | No |
| 24 | DUT |  |  | 2640 | -0.450 | -0.6041 | No |
| 25 | PRC1 |  |  | 2705 | -0.472 | -0.6142 | No |
| 26 | SHCBP1 |  |  | 2733 | -0.480 | -0.6133 | No |
| 27 | TUBA1B |  |  | 2785 | -0.498 | -0.6191 | Yes |
| 28 | HMGB1 |  |  | 2806 | -0.503 | -0.6158 | Yes |
| 29 | ARL6IP1 |  |  | 2823 | -0.510 | -0.6112 | Yes |
| 30 | CDKN2D |  |  | 2832 | -0.514 | -0.6042 | Yes |
| 31 | LIG1 |  |  | 2848 | -0.520 | -0.5992 | Yes |
| 32 | ECT2 |  |  | 2858 | -0.523 | -0.5923 | Yes |
| 33 | NUDT1 |  |  | 2859 | -0.524 | -0.5828 | Yes |
| 34 | TRIP13 |  |  | 2865 | -0.526 | -0.5747 | Yes |
| 35 | ANP32E |  |  | 2877 | -0.530 | -0.5682 | Yes |
| 36 | PTTG1 |  |  | 2895 | -0.540 | -0.5634 | Yes |
| 37 | FANCA |  |  | 2937 | -0.565 | -0.5651 | Yes |
| 38 | MZT1 |  |  | 2964 | -0.580 | -0.5621 | Yes |
| 39 | GGH |  |  | 2975 | -0.585 | -0.5544 | Yes |
| 40 | SMC2 |  |  | 2984 | -0.592 | -0.5460 | Yes |
| 41 | TCF19 |  |  | 3019 | -0.613 | -0.5448 | Yes |
| 42 | DNMT1 |  |  | 3035 | -0.622 | -0.5378 | Yes |
| 43 | NASP |  |  | 3106 | -0.682 | -0.5458 | Yes |
| 44 | DBF4 |  |  | 3117 | -0.690 | -0.5362 | Yes |
| 45 | PRR11 |  |  | 3125 | -0.698 | -0.5256 | Yes |
| 46 | SMC4 |  |  | 3126 | -0.699 | -0.5129 | Yes |
| 47 | CENPK |  |  | 3127 | -0.700 | -0.5002 | Yes |
| 48 | MCM5 |  |  | 3132 | -0.706 | -0.4885 | Yes |
| 49 | GMNN |  |  | 3140 | -0.719 | -0.4775 | Yes |
| 50 | CDK1 |  |  | 3149 | -0.727 | -0.4667 | Yes |
| 51 | CDT1 |  |  | 3179 | -0.758 | -0.4613 | Yes |
| 52 | MCM3 |  |  | 3182 | -0.761 | -0.4481 | Yes |
| 53 | BRCA1 |  |  | 3185 | -0.765 | -0.4348 | Yes |
| 54 | CIP2A |  |  | 3199 | -0.782 | -0.4244 | Yes |
| 55 | CDCA7 |  |  | 3203 | -0.785 | -0.4110 | Yes |
| 56 | BIRC5 |  |  | 3208 | -0.793 | -0.3978 | Yes |
| 57 | FBXO5 |  |  | 3212 | -0.797 | -0.3842 | Yes |
| 58 | DIAPH3 |  |  | 3213 | -0.800 | -0.3697 | Yes |
| 59 | HMGN2 |  |  | 3242 | -0.833 | -0.3627 | Yes |
| 60 | CENPM |  |  | 3269 | -0.881 | -0.3543 | Yes |
| 61 | CHAF1A |  |  | 3270 | -0.882 | -0.3383 | Yes |
| 62 | HELLS |  |  | 3284 | -0.901 | -0.3257 | Yes |
| 63 | RANBP1 |  |  | 3290 | -0.911 | -0.3106 | Yes |
| 64 | ZWINT |  |  | 3296 | -0.925 | -0.2953 | Yes |
| 65 | PCNA |  |  | 3319 | -0.963 | -0.2842 | Yes |
| 66 | UBE2T |  |  | 3330 | -0.979 | -0.2693 | Yes |
| 67 | EZH2 |  |  | 3342 | -1.015 | -0.2541 | Yes |
| 68 | DEK |  |  | 3344 | -1.025 | -0.2358 | Yes |
| 69 | HIST2H2AC |  |  | 3363 | -1.077 | -0.2215 | Yes |
| 70 | MCM7 |  |  | 3384 | -1.147 | -0.2065 | Yes |
| 71 | GINS2 |  |  | 3386 | -1.156 | -0.1858 | Yes |
| 72 | DHFR |  |  | 3387 | -1.159 | -0.1647 | Yes |
| 73 | HMGB2 |  |  | 3390 | -1.184 | -0.1438 | Yes |
| 74 | CLSPN |  |  | 3391 | -1.184 | -0.1223 | Yes |
| 75 | UBE2S |  |  | 3396 | -1.205 | -0.1016 | Yes |
| 76 | HIST1H1E |  |  | 3401 | -1.237 | -0.0803 | Yes |
| 77 | PCLAF |  |  | 3409 | -1.265 | -0.0594 | Yes |
| 78 | ATAD2 |  |  | 3415 | -1.278 | -0.0377 | Yes |
| 79 | HIST1H1C |  |  | 3427 | -1.335 | -0.0166 | Yes |
| 80 | HIST1H4C |  |  | 3493 | -2.248 | 0.0052 | Yes |
Table: GSEA details [plain text format]

  

Fig 2: NB\_KI\_CLUSTER5: Random ES distribution      
 Gene set null distribution of ES for **NB\_KI\_CLUSTER5**
